# Role of Visible Light-Activated Photocatalyst on the Reduction of Anthrax Spore-Induced Mortality in Mice

**DOI:** 10.1371/journal.pone.0004167

**Published:** 2009-01-09

**Authors:** Jyh-Hwa Kau, Der-Shan Sun, Hsin-Hsien Huang, Ming-Show Wong, Hung-Chi Lin, Hsin-Hou Chang

**Affiliations:** 1 Institute of Preventive Medicine, National Defense Medical Center, Taipei, Taiwan; 2 Institute of Molecular Biology and Human Genetics, Tzu-Chi University, Hualien, Taiwan; 3 Institute of Medical Science, Tzu-Chi University, Hualien, Taiwan; 4 Department of Materials Science and Engineering, National Dong-Hwa University, Hualien, Taiwan, Republic of China; Charité-Universitätsmedizin Berlin, Germany

## Abstract

**Background:**

Photocatalysis of titanium dioxide (TiO_2_) substrates is primarily induced by ultraviolet light irradiation. Anion-doped TiO_2_ substrates were shown to exhibit photocatalytic activities under visible-light illumination, relative environmentally-friendly materials. Their anti-spore activity against *Bacillus anthracis*, however, remains to be investigated. We evaluated these visible-light activated photocatalysts on the reduction of anthrax spore-induced pathogenesis.

**Methodology/Principal Findings:**

Standard plating method was used to determine the inactivation of anthrax spore by visible light-induced photocatalysis. Mouse models were further employed to investigate the suppressive effects of the photocatalysis on anthrax toxin- and spore-mediated mortality. We found that anti-spore activities of visible light illuminated nitrogen- or carbon-doped titania thin films significantly reduced viability of anthrax spores. Even though the spore-killing efficiency is only approximately 25%, our data indicate that spores from photocatalyzed groups but not untreated groups have a less survival rate after macrophage clearance. In addition, the photocatalysis could directly inactivate lethal toxin, the major virulence factor of *B. anthracis*. In agreement with these results, we found that the photocatalyzed spores have tenfold less potency to induce mortality in mice. These data suggest that the photocatalysis might injury the spores through inactivating spore components.

**Conclusion/Significance:**

Photocatalysis induced injuries of the spores might be more important than direct killing of spores to reduce pathogenicity in the host.

## Introduction

Naturally occurring anthrax is a disease acquired following contact with anthrax-infected animals or anthrax-contaminated animal products. The disease most commonly occurs in herbivores, which are infected by ingesting spores from the soil. For centuries, anthrax has caused disease in animals and, uncommonly, serious illness in humans throughout the world [1]. Research on anthrax as a biological weapon began more than 80 years ago [2]. Recently, the anthrax-letter attacks further evidenced this emerging terrorist threat, leading to renewed attention to the importance of prophylaxis, prevention and handling for anthrax [3]. Treatments or agents commonly cited to inactivate anthrax spores include heat, formaldehyde, hypochlorite solutions, chlorine dioxide, and radiation [4]. However, most of these treatments and reagents are hazardous to humans that limit their usage in public environments only after detecting the contamination sources, rather than prevention. Thus, a safer disinfection technique, which could exert a continuous antimicrobial effect in our living environment, would be highly desirable. Here, we present a visible light inducible photocatalyst which might provide a complementary and possibly alternative approach to meet this need.

Photocatalytic titanium dioxide (TiO_2_) substrates have been shown to eliminate organic compounds and to function as disinfectants [5]. Upon ultraviolet (UV) light excitation, the photon energy excites valence electrons and generates pairs of electrons and holes (electron-vacancy in the valence band) that react with atmospheric water and oxygen to yield reactive oxygen species (ROS) such as hydroxyl radicals ( **.** OH) and superoxide anions (O_2_^−^) [6]. Electron holes, **.** OH and O_2_^−^ are extremely reactive and could react with cellular components and function as biocides [5]. Since pure TiO_2_ photocatalyst is effective only upon irradiation by UV-light at levels that would induce serious damage to human cells, the potential applications of TiO_2_ substrates for use in our living environments are greatly restricted. Recently, anion-containing anatase TiO_2_ photocatalysts have been identified, which are activated by illumination with visible light [7], [8], offering the potential to overcome this problem [9]. Previously, we demonstrated that nitrogen-doped TiO_2_ [TiO_2_ (N)] photocatalyst could significantly eliminate *Escherichia coli* and other mild pathogens [9]. However, its antimicrobial activity and the antimicrobial mechanism against biological weapons, such as *Bacillus anthracis*, has not yet been reported. In this study, the anti-spore activity of TiO_2_ (N) photocatalysts against *B. anthracis* was compared with several other bacillus species including *Bacillus subtilis*, *Bacillus thuringiensis*, and *Bacillus cereus*. Among these bacteria, *B. subtilis* is broadly distributed worldwide where it mainly inhabits the upper layers of soil [10], [11]; *B. thuringiensis* is an insect pathogen [12]; and *B. cereus* causes broad clinical infections including local infections, septicemia, central nervous system infections, respiratory infections, endocarditis, pericarditis and food poisoning [13]. In this study, visible light-mediated photocatalysis was found to inactive 25%–40% spores of *B. subtilis, B. thuringiensis, B. cereus* and *B. anthracis*. Even though the efficiency of bacterial-killing is less than 1 log CFU, intriguingly, animal experiment revealed that the photocatalysis reduced more than ten times potency of *B. anthracis* spores to induce mortality in mice. To further investigate the underlining mechanism, here we analyzed photocatalyst-mediated inactivation of anthrax lethal toxin (LT) and the survival rate of spore after the macrophage clearance *in vitro*.

## Results

### UV-Vis absorption spectroscopic analysis

**Figure 1:**
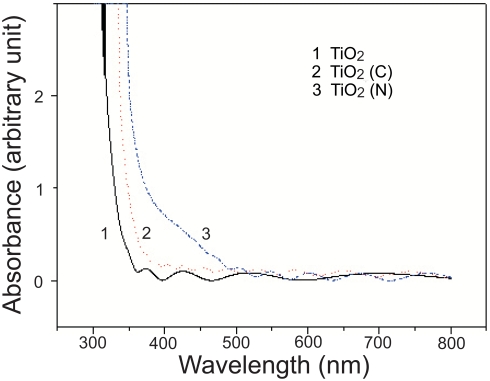
UV-Vis absorption spectrum analysis. UV-Vis absorption spectra of pure, carbon- and nitrogen-doped TiO_2_ thin films that used in this study were shown. Both doped samples absorbed light extending into the visible (>380 nm) region.

To investigate the physical properties of pure, carbon- and nitrogen- doped TiO_2_ films, respectively, X-ray diffraction (XRD) patterns and Raman spectra (data not shown) were obtained. Our results showed that all the films were anatase phase but the intensity of XRD and Raman peaks was slightly decreased in carbon and nitrogen doped films, indicating that the carbon/nitrogen incorporation induced decreasing crystallinity. The UV-Vis spectra of pure, carbon and nitrogen doped TiO_2_ films are shown in Fig. 1. The carbon and nitrogen substitution of oxygen in TiO_2_ caused the absorbance edge of TiO_2_ to shift to the higher wavelength region. The pure TiO_2_ film absorption edge which was at 380 nm gradually red-shifted to ∼425 nm and ∼565 nm in carbon and nitrogen doped TiO_2_ films, respectively. This shift in the visible region is the result of incorporation of carbon and nitrogen into the TiO_2_ network to form Ti-C and Ti-N bonds. Substitutional carbon or nitrogen atoms introduce new states (C2p or N2p) close to the valence band edge of TiO_2_ (i.e. O2p states). As a result of this the valence band edge shifts to higher energy compared with the reference TiO_2_ and the band gap narrows. The energy shift of the valence band depends on the overlap of carbon states and O2p states. A higher doping concentration of carbon or nitrogen results in higher energy shift due to significant overlap of carbon or nitrogen and oxygen states and this leads to a narrower band gap in the compound [14].

### Bactericidal activities of TiO_2_ photocatalysts against *Bacillus subtilis*

**Figure 2:**
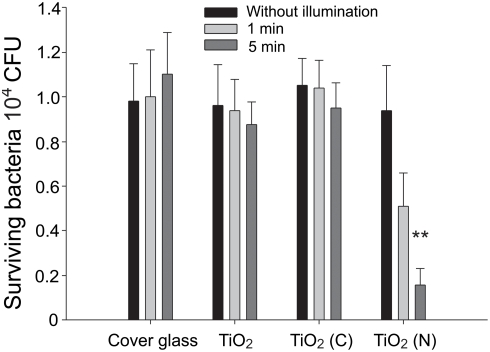
Bactericidal activity against *B. subtilis*. Visible-light induced bactericidal activities of TiO_2_–related substrates against *B. subtilis* after illumination at 4°C. Illumination was carried out at a light density of 3×10^4^ lux (90 mW/cm^2^) for either 1 or 5 min. “Without illumination” indicates experiments conducted in a dark room without illumination. **P<0.01, compared to both respective cover glass groups and without visible light illumination TiO_2_ (N) groups.

Before the spore experiments, living bacteria were used to test the photocatalysis system. Since *B. anthracis* is a hazardous microorganism, we first used *B. subtilis* as a surrogate to determine the bactericidal activity of nitrogen-doped [TiO_2_ (N)] and carbon-doped TiO_2_ [TiO_2_ (C)]. We placed 1×10^4^ CFU *B. subtilis* on different substrates including cover glass (silica, without TiO_2_ coating), and silica substrates coated with thin films of TiO_2_, TiO_2_ (N), and TiO_2_ (C). These preparations were then illuminated with visible light and the levels of surviving bacteria were quantified as previously described [9]. We found that TiO_2_ (N) exhibited a significantly better performance to reduce the number of surviving *B. subtilis* bacteria when compared to TiO_2_ and TiO_2_ (C) (Fig. 2, **P<0.01).

**Figure 3:**
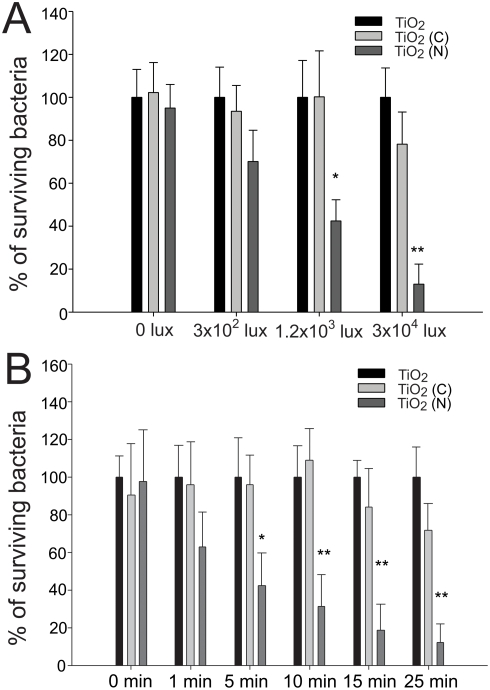
Dose dependency and kinetics. Dose dependent (A) and kinetic (B) analyses of the bactericidal activity of TiO_2_–related substrates against *B. subtilis* after visible light illumination were shown. Illumination was carried out either at different light densities for 25 min (A) or at a light density of 3×10^4^ lux (90 mW/cm^2^) for different time periods (B). For each illumination condition, the surviving bacteria on the TiO_2_ groups were normalized to 100%. *P<0.05 and **P<0.01 compared to the respective TiO_2_ groups.

To obtain dose dependent and kinetic data for *B. subtilis* on photocatalytic substrates, we further analyzed the effects of visible-light illumination at various distances (5 cm, 10 cm, 20 cm, and with respective illumination intensities of 3×10^4^, 1.2×10^3^, and 3×10^2^ lux) or at various time points (Fig. 3). The results showed that TiO_2_ and TiO_2_ (C) substrates had no detectable bacterial-killing effect, while TiO_2_ (N) contained significantly greater bactericidal activity, by which it induced nearly a 1 log CFU reduction under 3×10^4^ lux visible-light illumination for 25 minutes (Fig. 3A, 3B, *P<0.05; **P<0.01, compared to respective TiO_2_ groups). Although prolonged illuminations tended to increase the bactericidal effect of TiO_2_ (C) substrates (25 min, Fig. 3B), the killing efficiency was still not statistically significant as compared with the TiO_2_ groups.

### Anti-spore activities of TiO_2_ (N) against *Bacillus* species

**Figure 4:**
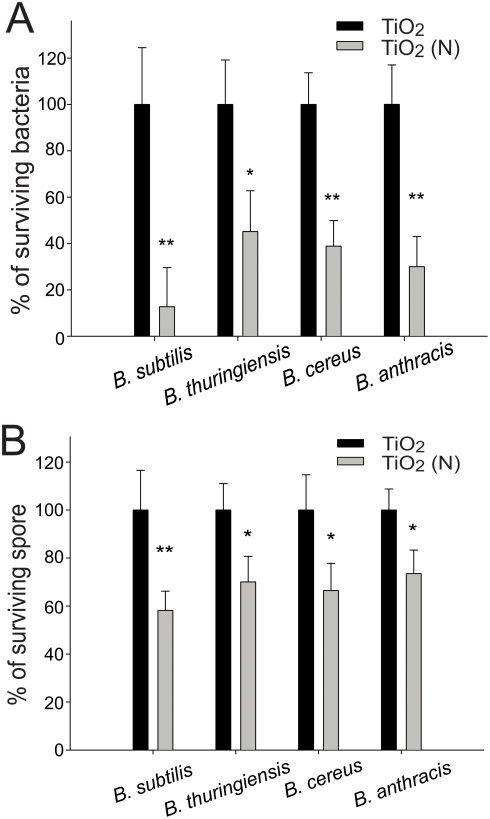
Elimination of living bacteria (A) and spores (B). Bacteria *B. subtilis*, *B. thuringiensis*, *B. cereus* and *B. anthracis* were placed on TiO_2_ and TiO_2_ (N) substrates, respectively. All surviving bacteria (A) or spores (B) in the TiO_2_ groups were normalized to 100%. The relative percentages of surviving pathogens in the TiO2 (N) groups are shown. The illumination intensity was 3×10^4^ lux (90 mW/cm^2^) and the reaction time was 25 minutes. *P<0.05 and **P<0.01 compared to respective TiO_2_ groups.

Photocatalyst-mediated killing was performed to determine the bactericidal effect of photocatalysis on *B. cereus, B. thuringiensis* and *B. anthracis*. Compared to TiO_2_ thin films, we found that TiO_2_ (N) thin films were significantly more effective in killing the living *B. cereus*, *B. thuringiensis* and *B. anthracis* bacteria under visible light illumination (Fig. 4A, *P<0.05, **P<0.01).

In spore experiments, TiO_2_ (N) also exhibited a better anti-spore activity than TiO_2_ thin films, although this activity was less efficient (25–40% killing/inactivation) (Fig. 4B, *P<0.05) compared to the results obtained in living bacteria experiments (Fig. 4A).

### Treatments of photocatalyzed spores and LT to mice

**Figure 5:**
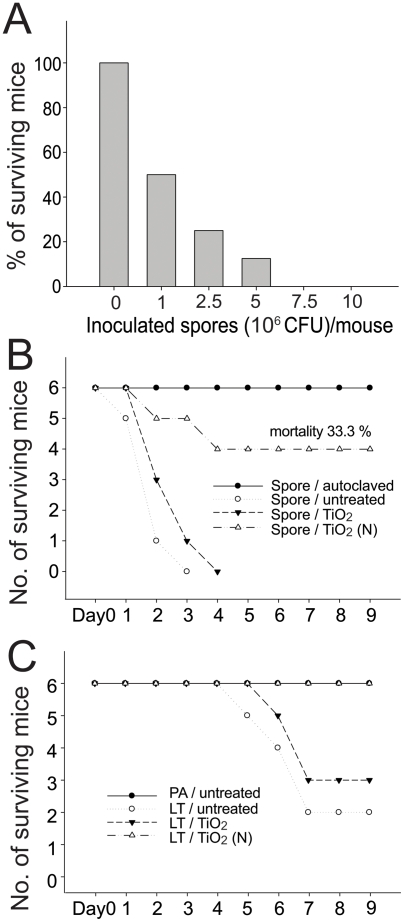
*B. anthracis* spore and LT caused mortality. Mortality of C57BL/6J mice after intravenous injection of different doses (0 to 1×10^7^ CFU) of *B. anthracis* spores within one-week interval is revealed (A) (n = 8). Aliquots of *B. anthracis* spores (1×10^7^ CFU) was subjected to photocatalysis on TiO_2_ and TiO_2_ (N) photocatalysts, respectively; spores in TiO_2_ (N) groups induced less mortality in mice (△) compared to untreated (○) or TiO_2_ (▾) groups (B) (n = 6). Aliquots of anthrax LT (500 µg PA : LF = 5∶1) was subjected to photocatalysis on TiO_2_ and TiO_2_ (N) photocatalysts, respectively; LT (100 µg/g) in TiO_2_ (N) groups (△) induced less mortality in mice compared to untreated (○) or TiO_2_ (▾) groups (C) (n = 6).

**Figure 6:**
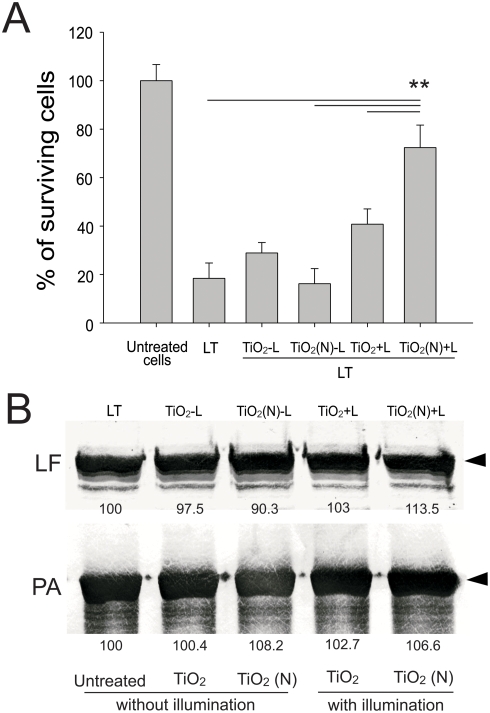
Cytotoxicity and Western blot analysis of photocatalyzed LT. Macrophage J774A.1 cells were subjected to LT treatments for three hours, surviving cells of untreated groups were adjusted to 100% (A). Columns designated TiO_2_ or TiO_2_ (N) represent that LT was pretreated with photocatalysis on TiO_2_ or TiO_2_ (N) substrates, respectively, before treated to J774A.1 cells. Columns designated “+L” or “−L” represent experimental conditions with or without light illumination, respectively. **P<0.01, compared to LT, TiO_2_-L and TiO_2_ (N)+L groups (A). Western blot measurements of intact LF and PA levels (arrows) of purified LT before and after visible light induced photocatalysis; band intensities of individual LF and PA samples are shown, and respective untreated groups were normalized to 100% (B).

A mouse model was further used to investigate whether these photocatalyzed spores are less pathogenic. Before the photocatalyst experiment, we determined anthrax spore mediated mortality in mice. We found that 50% mortality required a single inoculation of 1×10^6^ CFU anthrax spores, and the mortality reached to 100% when 7.5×10^6^ CFU spores were used (Fig. 5A). In photocatalyst experiment, we found that visible-light induced photocatalysis on TiO_2_ (N) but not pure TiO_2_ thin-films significantly attenuated the ability of anthrax spores (1×10^7^ CFU, before photocatalysis) to cause mortality in mice (Fig. 5B, n = 6). Notably, the mortality of mice in TiO_2_ (N) groups was lower than those given treatments of 1×10^6^ CFU spores without photocatalysis [Fig. 5B, TiO_2_ (N), mortality 33.3%, vs. Fig. 5A, 1×10^6^ groups, mortality 50%]. According to the killing efficiency estimated in spore-killing experiments (approximately 25%, Fig. 4B), around 7.5×10^6^ CFU spores should be remained viable, and theoretically the mortality of mouse should have reached up to 100% (Fig. 5A, 7.5×10^6^ CFU groups). As a result, the reduced pathogenicity of photocatalyzed spores could not be simply attributed to the reduction of viable spores. To explain this phenomenon, we hypothesized that photocatalysis on TiO_2_ (N) might not only eliminate 25% of the viable population but also injure the remaining spores through inactivating bacterial components. Such injuries could be resolved during germination of spores on cultural dishes but the repair process could not be accomplished in time to enable the bacteria escape from phagocytic clearance in mice. To investigate whether photocatalysis could inactivate protein components of *B. anthracis*, anthrax lethal toxin (LT), an example of bacterial proteins and the major virulence factor, was subjected to visible light activated photocatalysis on TiO_2_ and TiO_2_ (N) substrates. As expected, compared to untreated LT, photocatalysis of LT on TiO_2_ (N) but not on TiO_2_ substrates significantly reduced the potency of LT to induce mortality in mice (Fig. 5C). To investigate whether LT reduced its cytotoxicity after photocatalysis, a cell culture model was used. We found that LT could induce significant cell death of macrophage J774A.1 cells before but not after being subjected to TiO_2_ (N)-mediated photocatalysis (Fig. 6A, **P<0.01). Western blot analysis revealed that the intact forms of both purified lethal factor (LF) and protective antigen (PA) molecules, two components of LT [15], [16], did not decrease after photocatalysis (Fig. 6B). These data might suggest that protein degradation dose not play major role in the inactivation of LT.

### *In vitro* phagocytic clearance analysis

**Figure 7:**
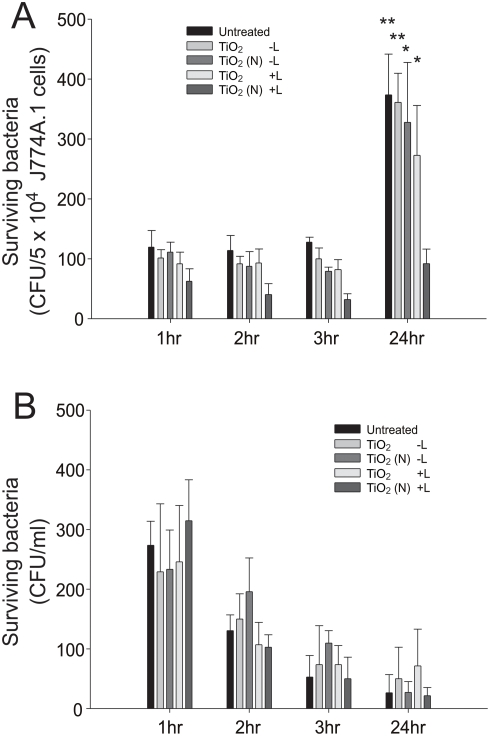
Surviving spores after clearance by macrophages. Anthrax spores were treated to J774A.1 macrophage cells (MOI: 0.01 spores/cell). Surviving bacteria (CFU) that harvested from macrophage cell lysate (A) or macrophage cell cultural medium (B) were shown. Columns designated TiO_2_ or TiO_2_ (N) represent anthrax spores were pretreated with photocatalysis on TiO_2_ and TiO_2_ (N) substrates, respectively. Columns designated “+L” or “−L” represent experimental conditions with or without light illumination, respectively. *P<0.05, **P<0.01, compared to 1 hr groups of respective conditions (A).

Anthrax spore can multiply in phagocytes [17]. To investigate whether photocatalysis might injure the spores and make them vulnerable for the clearance by phagocytes and further handicapped the bacterial amplification within phagocytes, photocatalyzed anthrax spores were then treated to macrophage J774A.1 cells. We found that spores in light illuminated-TiO_2_ (N) groups were not significantly multiplied in phagocytes within 24 hours (Fig. 7A, TiO_2_ (N)+L 1 hr vs. 24 hr). By contrast, untreated spores, or spores from groups without light illumination, or spores from illuminated-TiO_2_ groups were all significantly multiplied 3–4 fold within 24 hours (Fig. 7A, untreated/TiO_2_-L/TiO_2_ (N)-L/TiO_2_+L, 1 hr vs. 24 hr, *P<0.05, **P<0.01). Since low level of surviving bacteria in the 24-hour groups might be also possible attributed to the low phagocytic efficiency, to investigate whether photocatalysis might make these spores hard to be engulfed by phagocytes, we analyzed the remaining viable spores in the macrophage-culture medium. We found that the viable spores in the medium showed no significantly differences between each groups (Fig. 7B, 1 hr vs. 2 hr, 3 hr and 24 hr, all groups compared to each other), indicating that the low level of surviving bacteria in the TiO_2_ (N)+L/24 hr groups is not attributed to the low phagocytic efficiency (Fig. 7A). These results suggest that visible-light induced photocatalysis on TiO_2_ (N) substrates handicapped the amplification of anthrax in phagocytes.

## Discussion

The antibacterial property of photocatalysts was primarily induced under UV irradiation [5], [18], [19], and more recently by visible-light illuminated conditions [9], [20]–[25]. These studies provide valuable observations for the bactericidal activity of photocatalysts. Since these researches were mainly using laboratory *E. coli* strains as experimental materials, the potential application to apply on the eradication of spores of pathogenic bacteria, and especially the capability and the mechanism to reduce their pathogenicity were rarely discussed. Here we used spore forming bacillus bacteria as model systems to study the anti-spore activity of the visible-light photocatalyst. Since *B. anthracis* is a hazardous microorganism, in this study we first used *B. subtilis*, a bacillus bacterium with natural habitat in the soil that is not harmful to humans, as a surrogate for *B. anthracis*. We found that TiO_2_ (N) exerted anti-spore effects against *B. subtilis* and all our tested *Bacillus* species including *B. cereus*, *B. thuringiensis* and *B. anthracis*, with similar killing-efficiencies among these bacteria. These results suggest that *B. subtilis*, *B. cereus*, *B. thuringiensis* might be useful as surrogates for further photocatalyst-mediated anti-anthrax research. Even though the spore-killing efficiency is not good enough as compared with other well-developed methods [4], the contrast between the low spore-killing and a relatively high reduction of pathogenicity, inspired us to further investigate the underlining mechanism, by which it led us to find that the photocatalysis could inactivate the LT and handicapped the spore to multiply in the phagocytes. This is a finding not yet been reported previously.

The molecular target of photocatalyst-induced damage on bacteria is rarely discussed. It is shown that bacterial membrane lipid components likely to be the cellular target of photocatalyst induced ROS [5]. Using living *E. coli* as a model system, the authors found that TiO_2_-mediated photocatalysis promoted peroxidation of the polyunsaturated phospholipid component of bacterial membranes and then further led to respiratory activity loss and cell death [5]. Unlike vegetative cells, spores contain strong resistance to almost all antibacterial agents [4], [26], [27], as first observed by Koch over 100 years ago that *B. anthracis* spores could survive boiling. In the century that followed, it was learned that the protein components involving not only the composition of high density spore coat, a multilayered structure surrounding the spore that attributed to the resistance, but also the sensing responses to the renewed presence of nutrients in the environment, the condition under which the spore can convert to a growing cell through a process called germination [17], [27], [28]. Thus, the inactivation of a spore might be not just by disrupting the lipid components; deteriorated protein function could be involved as well. Photocatalysis-mediated protein dysfunction is rarely discussed. Evidence from enzyme-linked immunosorbent assays (ELISA) indicates that photocatalysis could affect the antigenic property of hepatitis B virus surface antigen and reduce its binding to specific antibody [29]. In this present study, we demonstrate the first time that photocatalysis could inactivate the bacterial exotoxin LT efficiently. Anthrax LT is a major virulence factor beneficial for the bacterium to establish initial infections in macrophages [30], [31]. Our Western blotting analysis revealed that both PA and LF, two components of LT, remained intact after photoreactions, indicating protein degradation does not play a major role in the inactivation. Although evidences indicate that LT is not expressed in anthrax spores [32], [33], since both protein toxin and spores are sensitive to photocatalysis, it seems likely that some of the spore proteins might be also sensitive to the photoreactions. Further investigation to identify the specific protein is needed.

Taken together, this study demonstrated that TiO_2_ (N) substrates could inactivate both spores and toxin of *B. anthracis* under illumination by ordinary light sources such as incandescent lamps. Our results suggest that the suppressed amplification of *B. anthracis* in phagocytes might be more important than the direct killing for photocatalysts to reduce the pathogenicity of the spores. These concepts might provide a new prospect to develop next generation antimicrobial agents.

## Materials and Methods

### Preparation of TiO_2_ substrates

Three types of films, TiO_2_, TiO_2−x_C_x_ and TiO_2−x_N_x,_ were prepared in an ion-assisted electron-beam evaporation system (Branchy Vacuum Technology Co., Ltd., Taoyuan, Taiwan). The distance between the rotating substrate holder and the electron-beam evaporation source was 550 mm. The chamber was evacuated with a mechanical pump (ALCATEL-2033SD, LACO Technologies, Salt Lake City, UT, USA) and a cryopump (Cryo-Torr8®, ULVAC Cryogenics, Chigasaki City, Kanagawa Prefecture, Japan) to a base pressure below 2.7×10^−4^ Pa. The substrates used were polished Si (100), quartz and glass coupons, which were sputter-etched with argon ions (Ar^+^) for 5 minutes prior to the deposition to remove any residual surface pollutants. The substrate temperature was maintained at 300°C with a quartz lamp. The TiO_2_ films were deposited in oxygen atmosphere (6.7 ×10^−3^ Pa) using rutile TiO_2_ (99.99%) as a source material. The nitrogen flow for TiO_2−x_N_x_ films was 15 standard cm^3^ min^−1^ through the ion gun at a constant pumping speed and the chamber pressure was 4.4 ×10^−2^ Pa. The carbon dioxide gas flow for TiO_2−x_C_x_ films was 7 standard cm3 min-1 and the chamber pressure was 2.6×10^−2^ Pa. The ion gun beam current of 10 mA and voltage of −1000 V was maintained by a Commonwealth Scientific IBS controller. Sufficient energy and current of the ion beam are critical to incorporate significant dopant concentration in the film. Without ion bombardment, it is difficult for the dopant to compete with the oxygen for incorporation into anatase titania. The deposition rate was adjusted to 0.2 nm·s^−1^ using a quartz crystal monitor for all films deposited at a thickness of 1.2 µm. The three types of films were prepared under the optimized conditions for their categories of anatase crystallinity and dopant concentration [34], [35]. The structure and crystallinity of the films were investigated using a Rigaku D/MAX-2500V 18 kW low angle X-ray diffractometer (XRD) (Rigaku, Shibuya-Ku, Tokyo, Japan) operating with Cu-K_α_ radiation at 40 kV and 150 mA and a Renishaw 1000B Raman spectrometer equipped with a charge-coupled detector (CCD) and a CW 532 nm wave length diode pump solid state (DPSS) laser as the excitation source (Renishaw plc, Representative Office, Nantun District, Taichung, Taiwan). The UV-Vis absorption spectra were recorded on a Hitachi 3300H spectrophotometer (Hitachi Taiwan, Taipei, Taiwan).

### Bacterial strains and culture

*B. anthracis* (ATCC 14186), which contains both pXO1 and pXO2 plasmids that express functional lethal toxin (LT) and edema toxin (ET), was grown and maintained as previously described [16], [36], [37]. *B. cereus* (ATCC 13061) and *B. thuringiensis* (ATCC 35646) were maintained and cultured in nutrient agar or nutrient broth at 30°C [38], [39], and *B. subtilis* (ATCC 39090) was maintained and cultured in trypticase soy agar or broth at 37°C [40]. All bacteria were stored in 50% medium and 50% glycerol solution in freezers at −80°C before use. To reactivate bacteria from frozen stocks, 25 µl bacterial stock solution was transferred to a test tube containing 5 ml of freshly prepared culture medium and then incubated at 30°C or 37°C under agitation overnight (16–18 hr). Spores of *B. anthracis* were prepared as previously described [41], [42]. Overnight tryptic soy broth cultures of *B. anthracis* were diluted to about 10^7^ CFU/ml in phosphate-buffered saline, and 0.1-ml aliquots were inoculated onto blood agar plates. The agar plates were incubated at 25–37°C until 90–99% phase-bright spores were observed by phase-contrast light microscopy (see below). Spores were harvested and washed with cold sterile distilled ionized (DI) water as previously described [41] and stored in DI water at 4°C until use for up to 2 weeks, changing the water at least once a week, or in the freezer at −20°C for up to a month. The quality of spores was determined by two complementary criteria previously established to validate the presence of dormant spores [42]. The criteria consisted in the evaluation of (i) the absence of vegetative cells (rods) determined by microscopic examination as described, and (ii) the survival of spores in hydrochloric acid (2.5 N). Spore preparations of *B. subtilis*, *B. cereus* and *B. thuringiensis* were followed a similar protocol.

### Photocatalytic reaction and detection of viable bacteria

In this study, bacterial concentrations were either determined by the standard plating method or inferred from optical density readings at 600 nm (OD_600_). For each *Bacillus* species, a factor for converting the OD_600_ values of the bacterial culture to concentration (CFU/ml) was calculated as follows. A fresh bacterial culture was diluted by factors of 10^−1^ to 10^−7^, and OD_600_ of these dilutions was measured. Bacterial concentrations of these dilutions were determined by the standard plating method. The OD_600_ values were plotted against the bacterial concentration log values, and the conversion factors for the particular bacteria were calculated from three independent measurements. For example, the conversion factor for *B. subtilis* was calculated to be 1×10^8^ CFU/ml per OD_600_ by this method.

In order to determine the bactericidal effects of the TiO_2_-related substrates, 200 µl of bacterial overnight culture was transferred into 5 ml of culture medium and incubated at 37°C until an OD_600_ of 0.3 to 0.6 (log phase) was reached. The bacterial concentrations were calculated using the previously determined conversion factor for the bacteria, and the cultures were diluted to 1×10^5^ CFU/ml with culture medium. One hundred microliters (1×10^4^ CFU) was then applied to an area of approximately 1 cm^2^ of the different TiO_2_-related substrates using a plastic yellow tip. The bacteria substrates were then placed under an incandescent lamp (Classictone incandescent lamp, 60W, Philips, Taiwan) for photocatalytic reaction. A light meter (model LX-102, Lutron Electronic Enterprises, Taiwan) was used to record the illumination density. In the following photocatalysis experiments, the bacteria solution was supplied by 5–10 µl additional distil water every 5 minutes to maintain approximately 100 µl of total volume. After the photocatalyst-killing for 25 minutes, the bacteria containing solution approximately 85 µl was recovered from the photocatalyst substrates using a tip, additional 60 µl fresh medium was used to wash the remaining bacteria on the photocatalyst substrates. Two bacterial containing solutions were mixed, diluted and placed on agar plates. To test whether spores have different efficiency to adhere on TiO_2_ substrates that might influence the photocatalysis result, we analyze the spore recovery rates from TiO_2_, TiO_2_ (C) and TiO_2_, TiO_2_ (N) substrates. We found that the recovery rates are similar (data not shown), which has no statistic significant among these groups. In the dose-dependence experiments, illumination was carried out for 5 min at distances of 5, 10, and 15 cm from the lamp, corresponding to the illumination densities of 3×10^4^, 1.2×10^3^, and 3×10^2^ lux (lumen/m^2^)(90, 30, and 10 mW/cm^2^), respectively. In the kinetic analysis experiments, illumination was carried out for 1, 5, 10, 15, and 25 min at a distance of 5 cm, corresponding to an illumination density of 3×10^4^ lux (90 mW/cm^2^). Unless specified, illumination was carried out in a 4°C cold room to prevent over-heating of the photocatalyst substrates and prevent drying. After illumination, the bacterial solutions were recovered from the photocatalyst substrates, and an aliquot of fresh culture medium was used to collect the residual bacteria on the substrates. The two bacterial solutions were pooled to make a total volume of 150 µl. The bacterial concentration was determined by the standard plating method immediately after the bacterial collection, and the percentage of surviving bacteria was calculated. In spore experiments, 1×10^4^ CFU (1×10^5^ CFU/ml in 100 µl) were used, and the procedures followed the same protocols as in the live bacteria experiments.

### Mouse model

Six to 8 week**-**old C57BL/6J mice were purchased from the National Experimental Animal Center (Taipei, Taiwan) [37], [43]. The methods used in bacteremia experiments were modified from previous descriptions [9]. Mortality of C57BL/6J mice from various anthrax spores treatments (from 1×10^6^ to 1×10^7^) was recorded within one week to serve as reference points. To determine the anti-bacterial effect of TiO_2_ (N), each mouse received an intravenous injection of 1×10^5^ CFU spores of *B. anthracis*, a lethal dose for mice, with or without pretreatment by photocatalysis on TiO_2_ (N) or TiO_2_ substrates (3×10^4^ lux, 10 minutes at 4°C). In the lethal toxin (LT) experiments, mortality of mice after a lethal dose of LT was performed based on previously described methods [37]. Each mouse received an intravenous injection of LT (100 µg/g, LF:PA = 1∶5), a lethal dose for mice, with or without pretreatment of photocatalysis on TiO_2_ (N) or TiO_2_ substrates (3×10^4^ lux, 10 minutes at 4°C or 25°C). The mortality of mice was then recorded. During the photocatalysis reaction, the distance between lamps with bacteria- or LT-containing photocatalyst substrates was 5 cm, corresponding to an illumination density of 3×10^4^ lux (or 90 mW/cm^2^). Relative protein levels of PA and LF in the LT mixtures used in animal experiments were detected by Western blot using rabbit polyclonal anti-PA and anti-LF antibodies, and then probed by secondary horseradish peroxidase-conjugated goat anti-rabbit immunoglobulins [16], [44], [45]. The gel intensities of PA and LF were measured using Image J software (version 1.32; National Institutes of Health, USA). The Animal Care and Use Committee of Tzu-Chi University approved the protocol of the mice experiments.

### Cytotoxicity analysis

Cytotoxicity of LT was measured following a previously described method [16]. In brief, a cytotoxic dose of LT (10 mg/L, LF:PA = 1∶5) with or without photocatalysis pretreatment on TiO_2_ and TiO_2_ (N) thin film was used to treat mouse macrophage J774A.1 cells. Three hours after the LT treatments, cell viability of J774A.1 cells were measured using a WST-1 kit (Roche, Mannheim, Germany), following the instructions provided by the manufacturer. Photocatalysis of purified LT was carried out as described in mouse experiments (3×10^4^ lux, 10 min at 4°C).

### Phagocytosis analysis

Anthrax spores is normal saline (100 µl, 1×10^5^ CFU/ml) were placed on cover glass, TiO_2_ and TiO_2_ (N) coated thin films [9], respectively. The spore-photocatalyst mixtures (100 µl) were then illuminated with visible light (Classictone incandescent lamp, 60W, Philips; 90 mW/cm^2^; lamp-target distance 10 cm) for 30 minutes. After illumination, the spore containing solutions (85 µl) were recovered from the photocatalyst substrates, and an aliquot of normal saline (60 µl) was used to collect the residual spores on the substrates. The two spore solutions were pooled to make a total volume of 145 µl. This spore solution was then added into one well of a six-well cell culture dish that containing confluent murine macrophage J774A.1 cells (1×10^6^ cells/well) (MOI: 0.01 spores/cell). After phagocytosis was carried out for one, two and three hours, respectively, culture medium was removed, and 200 µl cell lysis buffer (100mM Tris-HCl [pH 7.4], 10mM MgCl_2_, 100mM NaCl, 0.2% sucrose, 0.5% Triton X-100) that was modified from previous literatures [46], [47], was then added to release the cell-engulfed or cell-bound spores. Additional 100 µl fresh medium was used to further collect the residual spores on the dishes. Two spore containing solutions were mixed and placed on agar plates. Cell culture medium (DMEM) without antibiotics and serum supplements was used in this analysis.

### Statistical analysis

All results were calculated from data of at least three independent experiments. A *t*-test was used to assess the significance of differences in results of anti-microbial effects. A *P* value of less than 0.05 (*P*<0.05) was considered statistically significant. The statistical tests were carried out and output to graphs using Microsoft Excel (Microsoft Taiwan, Taipei, Taiwan) and SigmaPlot (Systat Software, Point Richmond, CA, USA) software.
